# Host Genetic Background Impacts Disease Outcome During Intrauterine Infection with *Ureaplasma parvum*

**DOI:** 10.1371/journal.pone.0044047

**Published:** 2012-08-29

**Authors:** Maria von Chamier, Ayman Allam, Mary B. Brown, Mary K. Reinhard, Leticia Reyes

**Affiliations:** 1 Animal Care Services, University of Florida, Gainesville, Florida, United States of America; 2 Department of Infectious Diseases and Pathology and D. H. Barron Reproductive and Perinatal Biology Research Program, University of Florida, Gainesville, Florida, United States of America; Institut Jacques Monod - UMR 7592 CNRS - Université Paris Diderot, France

## Abstract

*Ureaplasma parvum,* an opportunistic pathogen of the human urogenital tract, has been implicated in contributing to chorioamnionitis, fetal morbidity, and fetal mortality. It has been proposed that the host genetic background is a critical factor in adverse pregnancy outcome as sequela to *U. parvum* intra-amniotic infection. To test this hypothesis we assessed the impact of intrauterine *U. parvum* infection in the prototypical TH1/M1 C57BL/6 and TH2/M2 BALB/c mouse strain. Sterile medium or *U. parvum* was inoculated into each uterine horn and animals were evaluated for intra-amniotic infection, fetal infection, chorioamnionitis and fetal pathology at 72 hours post-inoculation. Disease outcome was assessed by microbial culture, *in situ* detection of *U. parvum* in fetal and utero-placental tissues, grading of chorioamnionitis, and placental gene expression of IL-1α, IL-1β, IL-6, TNF-α, S100A8, and S100A9. Placental infection and colonization rates were equivalent in both strains. The *in situ* distribution of *U. parvum* in placental tissues was also similar. However, a significantly greater proportion of BALB/c fetuses were infected (P<0.02). C57BL/6 infected animals predominantly exhibited mild to moderate chorioamnionitis (P<0.0001), and a significant reduction in placental expression of IL-1α, IL-1β, IL-6, TNF-α, S100A8, and S100A9 compared to sham controls (P<0.02). Conversely, severe protracted chorioamnionitis with cellular necrosis was the predominant lesion phenotype in BALB/c mice, which also exhibited a significant increase in placental expression of IL-1α, IL-1β, IL-6, TNF-α, S100A8, and S100A9 (P<0.01). Fetal pathology in BALB/c was multi-organ and included brain, lung, heart, liver, and intestine, whereas fetal pathology in C57BL/6 was only detected in the liver and intestines. These results confirm that the host genetic background is a major determinant in ureaplasmal induced chorioamnionitis with fetal infection and fetal inflammatory response.

## Introduction

Chorioamnionitis, an inflammatory disorder of fetal membranes, is an underlying pathologic process of complicated pregnancies such as premature rupture of membranes (PROM), spontaneous preterm labor (PTL), fetal inflammatory response syndrome, and fetal death [1], [2], [3], [4]. Although CAM has multiple etiologies, microbial invasion of the amniotic cavity (MIAC) is the most common cause of this pathologic process [3], [5]. *Ureaplasma parvum* and *U. urealyticum* are the most common bacteria associated with CAM and its perinatal sequela [5], [6], [7], [8]. Both *U. urealyticum* and *U. parvum* have been identified in amniotic fluid [9], [10], [11], [12], [13], fetal cord blood [14], fetal and neonatal lung [15], [16], cerebrospinal fluid [8], [17], and fetal gastro-intestinal aspirates [18], [19]. Various studies with animal models have demonstrated a causal relationship between monotypic intrauterine infection with *Ureaplasma* species and spontaneous preterm delivery [20], neonatal bronchopulmonary disease [21], [22], [23], [24], [25], antenatal brain injury [26], and fetal dermatitis [27]. However, the pathogenesis of ureaplasmal induced chorioamnionitis and adverse pregnancy outcome is not yet clearly elucidated. For example, *Ureaplasma* species or serovar type is not a critical factor in whether or not intrauterine infection remains as uncomplicated asymptomatic infection or proceeds to severe chorioamnionitis with adverse pregnancy outcome [28], [29], [30]. Moreover, recent studies that focused on the role of *Ureaplasma* multiple banded antigens in the pathogenesis of chorioamnionitis suggest that ureaplasmas themselves are not intrinsically avirulent or virulent, but rather, the host’s immune response to the presence of the bacterium within the amniotic cavity is the predominant determinant in disease pathogenesis [31].

Pathologic features of chorioamnionitis include inflammatory cell infiltrates (neutrophils) with other features of inflammation such as edema, fibrin deposition and necrosis of neutrophils and amniotic epithelial cells [3], [32]. When the fetus is affected, inflammatory lesions can also be observed in the umbilical cord [2], [32]. Elevated concentrations of IL-1β, IL-6, IL-8, and TNF-α are usually found within the amniotic fluid of patients with chorioamnionitis and/or funisitis [1], [2], [33]. Increased levels of calgranulins S100A8, S100A9, and S100A12 have also been detected in the amniotic fluid of chorioamnionitis patients experiencing PTL and/or fetal inflammatory response syndrome [33], [34], [35]. Although not considered a characteristic of histologic chorioamnionitis, decidual macrophages are also important in the disease process since they are early initiators of the host inflammatory response to bacterial invasion; they also regulate the progression or resolution of chorioamnionitis [36].

It is well accepted that exaggerated local inflammation in response to intrauterine infection will trigger PROM, PTL, and/or fetal inflammatory response syndrome [4], and the host response to infection may be influenced by host genetic factors [1]. For example, there are racial disparities in the inflammatory response associated with PTL such as over production of IL-6 and IL-8 in Caucasian women versus an increase in IL-1β and TNF-α in African American women [37], [38]. These differences have been found to correlate with variations in single nucleotide polymorphisms (SNP) that are associated with each race [39], [40]. Polymorphism in the promoter region of TNF-α gene has been documented to be risk factor for PTL in the presence of bacterial vaginosis [41]. Thus, a host’s genetic predisposition to react to infection with an intense inflammatory response is likely to be a significant factor in the development of adverse pregnancy outcomes during intrauterine infection with *Ureaplasmas.*

C57BL/6 and BALB/c mice have genetically distinct immunologic phenotypes that have been used to elucidate innate immune responses that confer resistance or susceptibility to infection and disease. In addition to their distinct TH1/TH2 cytokine profiles, C57BL/6 and BALB/c mice also display functional differences in toll-like receptor responsiveness, and differences in macrophage and neutrophil reactivity that have been shown to influence disease outcomes during infections caused by *Trypanosoma cruzi, Leishmania major,* and sepsis [42], [43], [44], [45], [46]. Since these innate immune responses are involved in the pathogenesis of chorioamnionitis and adverse pregnancy outcomes, we assessed the impact of *U. parvum* intrauterine infection in both C57BL/6 and BALB/c mice. Despite both strains having similar microbial load, BALB/c mice were more susceptible to fetal infection than C57BL/6. Furthermore, chorioamnionitis was more severe in BALB/c mice.

## Results

### BALB/c Mice are More Susceptible to Fetal Infection with *U. parvum*

*U. parvum* colonization of fetal and placental tissues was evaluated at gestation day (GD) 17, which was 72 hours post-inoculation. All tissues from sham inoculated animals were culture negative for *U. parvum*. Placental infection rates among C57BL/6 mice were 100% and for BALB/c mice, 92%. Mouse strain did not have an impact on the microbial load of *U. parvum* within placental tissues. For example, the mean ± SD log CFU of *U. parvum* was 3.46±1.74 from infected C57BL/6 and 3.53±1.05 from BALB/c placental tissues. The microbial load in infected fetal tissues was also similar for both strains. In C57BL/6 the log CFU of *U. parvum* isolated from infected fetuses was 3.75±1.89 (mean ± SD) and in BALB/c fetuses it was 2.31±1.65. However, the frequency of fetuses found to be infected in inoculated dams was significantly different between the two mouse strains (P<0.02 by Chi square). Specifically, only 6 of 32 (18.8%) C57BL/6 fetuses were infected whereas 18 of 38 (56%) BALB/c fetuses were infected at 72 hours post-infection.

**Figure 1:**
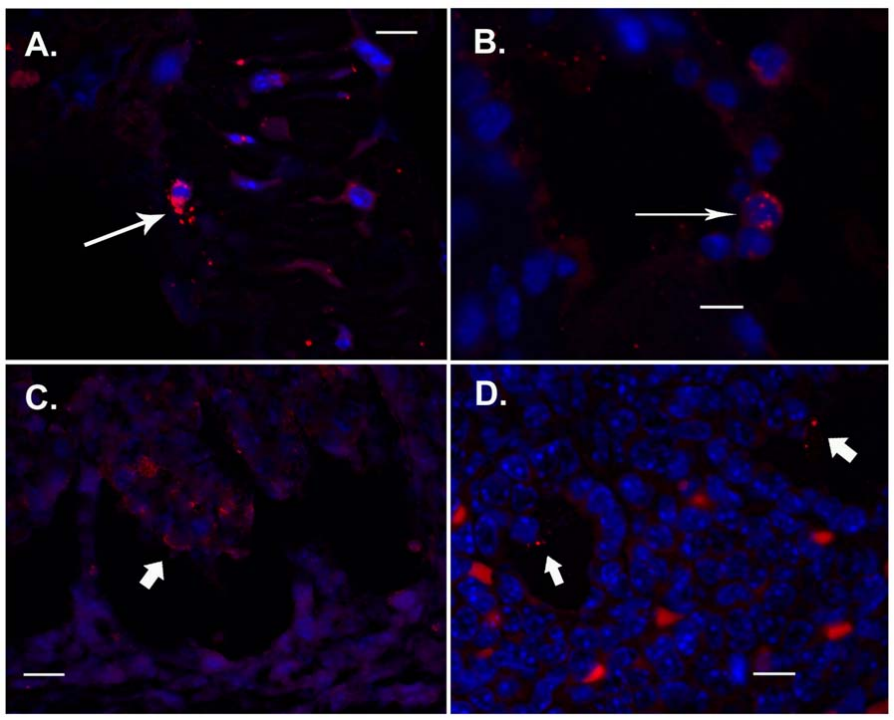
*In situ* detection of *U. parvum* in placental and fetal tissues. Representative 2D projection images (600 x magnifications) of placental choriodecidual junction (A), vitelline membrane (B), fetal intestine (C), and fetal lung (D). Scale bars are equivalent to 100 µm. *U. parvum* (red) was initially labeled with rabbit polyclonal antibody as previously described [69], [71]. Cell nuclei (blue) were labeled with DAPI. Long arrows (A and B) are highlighting *U. parvum* engulfed by polymorphonuclear cells. Block arrows (D and E) are highlighting epicellular *U. parvum*.

Given the discrepancy in the rate of fetal colonization between C57BL/6 and BALB/c mice, we next assessed the *in situ* location of *U. parvum* within their intrauterine and fetal tissues by immunofluorescent microscopy (Figure 1, isotype controls can be viewed in file S1). Regardless of fetal infection status, clusters of *U. parvum* were identified in the chorionic plate, choriodecidual junction of the placental membranes, the chorioamnion and vitelline membranes in both C57BL/6 and BALB/c mice (Figure 1). In some sections *U. parvum* organisms could be found engulfed by polymorphonuclear cells (Figure 1A and B). In fetal tissues, *U. parvum* organisms were identified within the fetal lung of one BALB/c mouse, and in the intestinal lumen of both BALB/c and C57BL/6 fetuses (Figure 1C and D).

### C57BL/6 and BALB/c Mice Exhibit Divergent Inflammatory Responses to Intrauterine Infection with *U. parvum*

**Figure 2:**
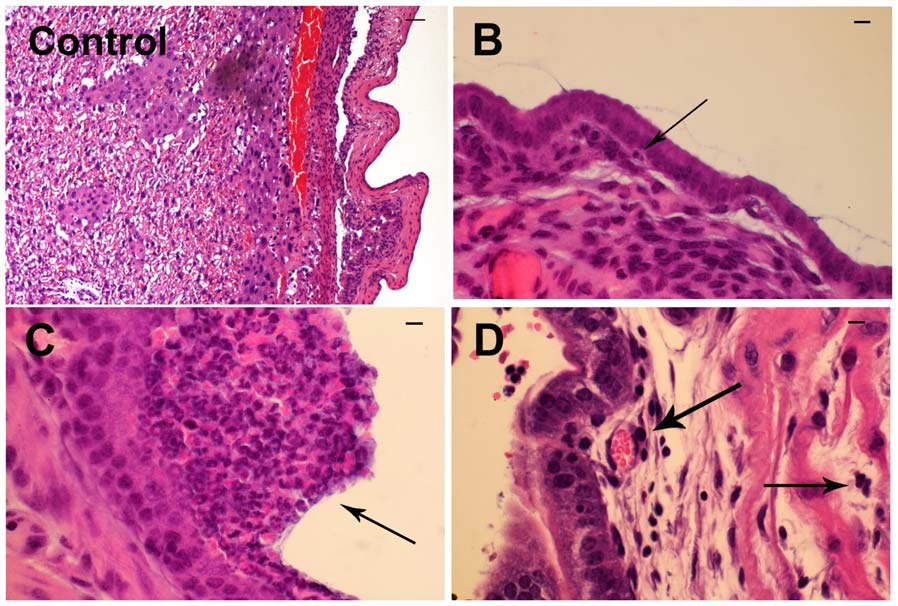
Representative images of metritis. Sham inoculated (Control) and representative lesions in *U. parvum* infected C57BL/6 mice (B, mild metritis) and in *U. parvum* infected BALB/c mice (C and D, more extensive metritis). Scale bars are equivalent to 100 µm. Arrows are highlighting neutrophilic infiltrates.

**Figure 3:**
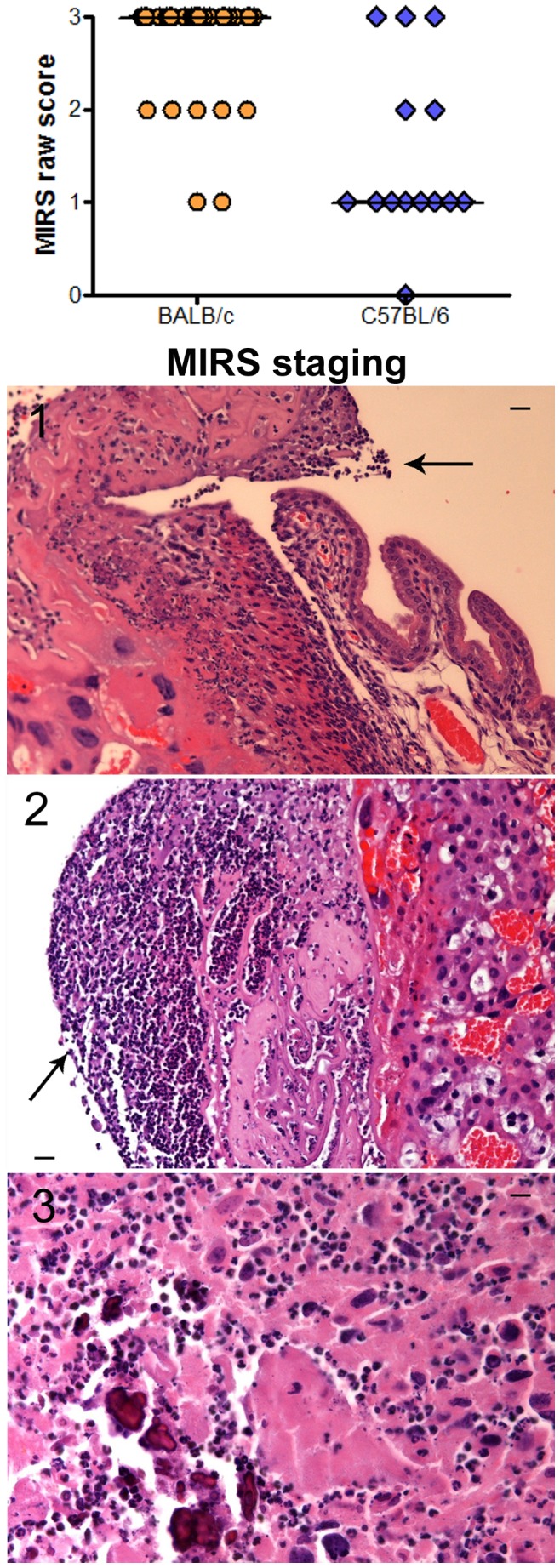
Maternal inflammatory response defined by the degree of chorioamnionitis in *U. parvum* infected BALB/c and C57BL/6 dams. Distribution of raw maternal inflammatory response (MIR) placental scores using the scoring criteria of Redline et al. [32] summarized in Table 1. Median scores are demarcated with a horizontal line in each graph. Data are a compilation of 3 independent experiments. Accompanying H & E stained photomicrographs of MIRS stages 1–3. Scale bars are equivalent to 100 µm. Arrows are highlighting neutrophilic infiltrates.

We next evaluated the maternal and fetal inflammatory response to *U. parvum* by histopathology. The maternal inflammatory response was assessed by two methods. The first was a qualitative assessment of localized metritis in the portion of the uterine tissue attached to the placenta and the uterine tissue surrounding the implantation site (Figure 2). The second approach employed an established scoring system for measuring the maternal contribution to chorioamnionitis as defined by Redline [3], [32] and summarized in Table 1. Most C57BL/6 dams exhibited metritis that was characterized as scant neutrophilic infiltrates in the endometrium and myometrium (Figure 2B). The majority of BALB/c dams exhibited extensive metritis in which dense neutrophilic infiltrates were observed in the endometrium and myometrium (Figures 2C and D). With regard to chorioamnionitis, the predominant lesion detected in C57BL/6 placentas was a patchy influx of neutrophils in the subchorionic plate or the choriodecidual junction of the placental membranes (stage 1, Figure 3). However, in BALB/c mice, the predominant placental lesion was necrotizing chorioamnionitis (stage 3, Figure 3) with desquamation of amnion epithelial cells (P<0.0001).

**Table 1:** Lesion scoring criteria for maternal inflammatory response [32].

Stage Score	Diagnosis	Lesion Description
**0**	Normal	None
**1**	Acute subchorionitis or acutechorionitis	A diffuse band of neutrophils either below the chorionic plate (acute subchorionitis) or at the choriodecidual junction of the placental membranes (acute chorionitis).
**2**	Acute chorioamnionitis	Breach of maternal-fetal barrier at the chorion to enter the connective tissue of the chorioamnion.
**3**	Necrotizing chorioamnionitis	Neutrophil degradation and fragmentation, thickening of amniotic basement membrane, and desquamation of amnionic epithelial cells involving ≥30% of the amniotic surface.

**Figure 4:**
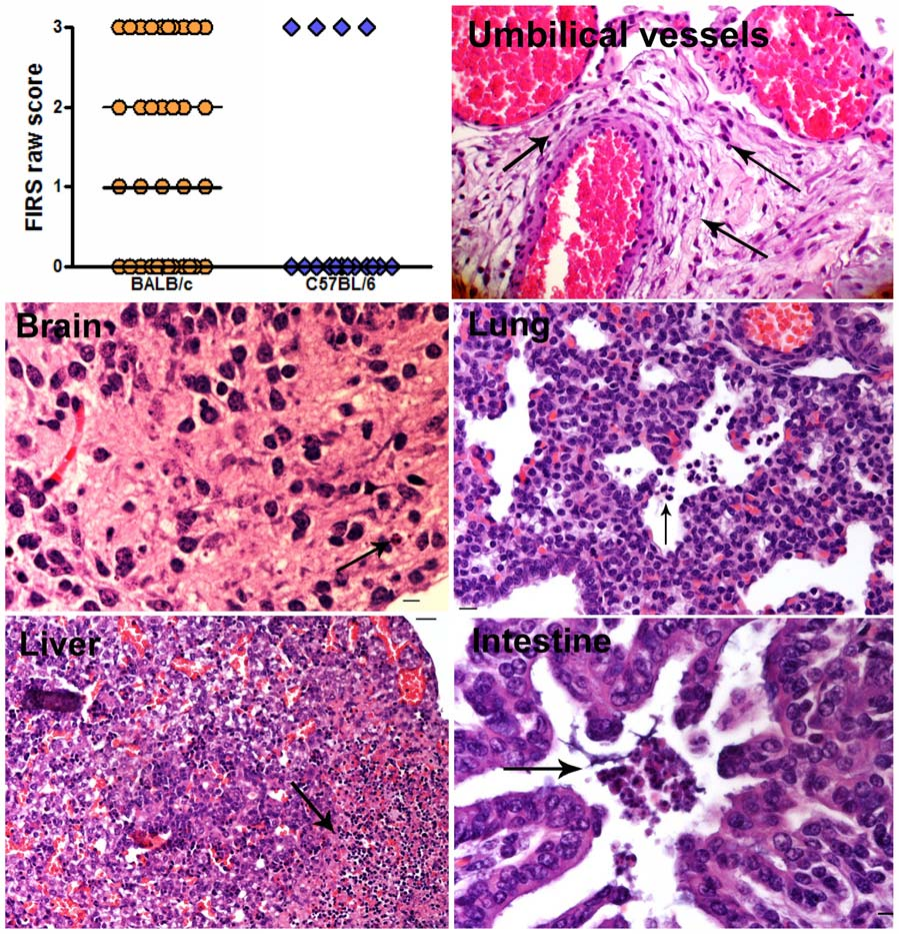
Fetal inflammatory response (FIR) in *U. parvum* infected BALB/c and C57BL/6 mice. Distribution of raw FIR scores as described in Table 2. Median scores are demarcated with a horizontal line in each graph. Data are a compilation of 3 independent experiments. Accompanying H & E stained sections are representative images of fetal pathology in umbilical vessels, brain, lung, liver, and intestine. Scale bar is equivalent to 100 µm. Arrows are highlighting neutrophilic infiltrates.

Fetal inflammatory responses (FIR) were assessed by using established criteria, which is summarized in Table 2 [32]. In addition, we modified this scoring system to include fetal lesions such as fetal necrosis, hepatitis, necrotizing enterocolitis, and pulmonary inflammation, which are lesions consistent with fetal inflammatory response syndrome or ureaplasmal associated fetal/neonatal morbidity such as enteritis, pneumonitis, and brain injury [2], [8], [47], [48], [49]. In general, FIR scores in BALB/c mice were higher than in C57BL/6 (Figure 4). Specifically, we observed more chorionic vasculitis and umbilical vasculitis in BALB/c placentas than in C57BL/6 placentas (P<0.003). Moreover, more BALB/c fetuses exhibited a wider range of pathology than C57BL/6 fetuses. For example, 7 of 8 BALB/c fetuses from 6 *U. parvum* infected dams had some degree of pathology. Of these, 2 fetuses had extensive necrosis, which made further assessment of organ pathology impossible. The remaining BALB/c fetuses exhibited multi-organ system involvement: encephalitis with pneumonitis, or myocarditis with hepatitis, or hepatic necrosis with enteritis. In the C57BL/6 group, only 4 of 12 fetuses obtained from 5 *U. parvum* infected dams had any evidence of pathology. One C57BL/6 fetus had extensive necrosis and 3 fetuses had hepatitis and/or enteritis.

**Table 2:** Fetal inflammatory response criteria modified from Redline 2006 [32].

Score	Stage	Histologic lesions
**0**	Normal	None
**1**	Chorionic vasculitis and/or umbilical phlebitis	Neutrophils in the chorionic vessels (chorionic vasculitis) and/or umbilical vein (umbilical phlebitis)
**2**	Umbilical arteritis	Entry of neutrophils into the wall of the umbilical artery, with or without minor degrees of extravasation
**3**	Necrotizing funisitis or necrotizing and inflammatory infiltrates in fetal organs	Organization of neutrophils in the umbilical vessels or necrotizing and inflammatory infiltrates infetal organs such as intestine, liver, brain, heart, or lung

Since BALB/c and C57BL/6 mice produce different cytokine responses during infection [43], [50], we profiled the placental inflammatory response in both sham inoculated and *U. parvum* infected animals by quantitative RT-PCR or ELISA. Our analysis included inflammatory mediators reported to be associated with chorioamnionitis, preterm labor, and fetal inflammatory response syndrome such as IL-1α, IL-1β, IL-6, TNF-α, S100A8, and S100A9 [1], [20], [31], [34], [49], [51], [52]. In addition, we evaluated the production of IL-10 since it has anti-inflammatory properties [53] and can be induced by fetal ovine skin in response to *U. parvum* infection [54].

**Figure 5:**
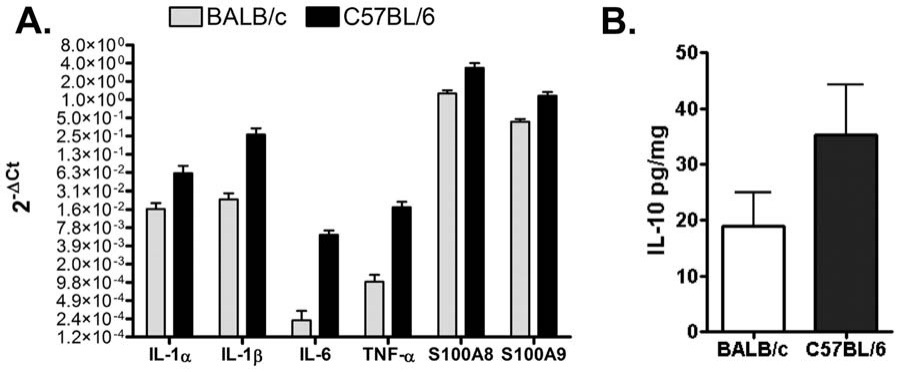
Profiling of placental inflammatory mediators in sham inoculated BALB/c and C57BL/6 mice. ( A) Normalized gene expression values are expressed as 2*^−^* ^Δ*Ct*^ [72]. (B) Production of IL-10 expressed as mean (pg/gm weight of tissue) ± SD. Values represent 5 biological replicates from 3 independent experiments.

We first evaluated mouse strain specific characteristics by comparing the inflammatory profiles of sham inoculated BALB/c to C57BL/6 animals (Figure 5). In uninfected animals, [55] the placental gene expression of IL-1α, IL-1β, IL-6, TNF-α, S100A8, and S100A9 was significantly higher in C57BL/6 than in BALB/c mice (P<0.0005, Figure 5A). The most dramatic differences were observed in C57BL/6 expression of IL-1β, IL-6, and TNF-α, which exceeded expression levels in all other treatment groups including *U. parvum* infected tissues from both mouse strains (P<0.001, data not shown). Placental tissues from C57BL/6 animals also produced more IL-10 than BALB/c mice (Figure 5B, P<0.03).

**Figure 6:**
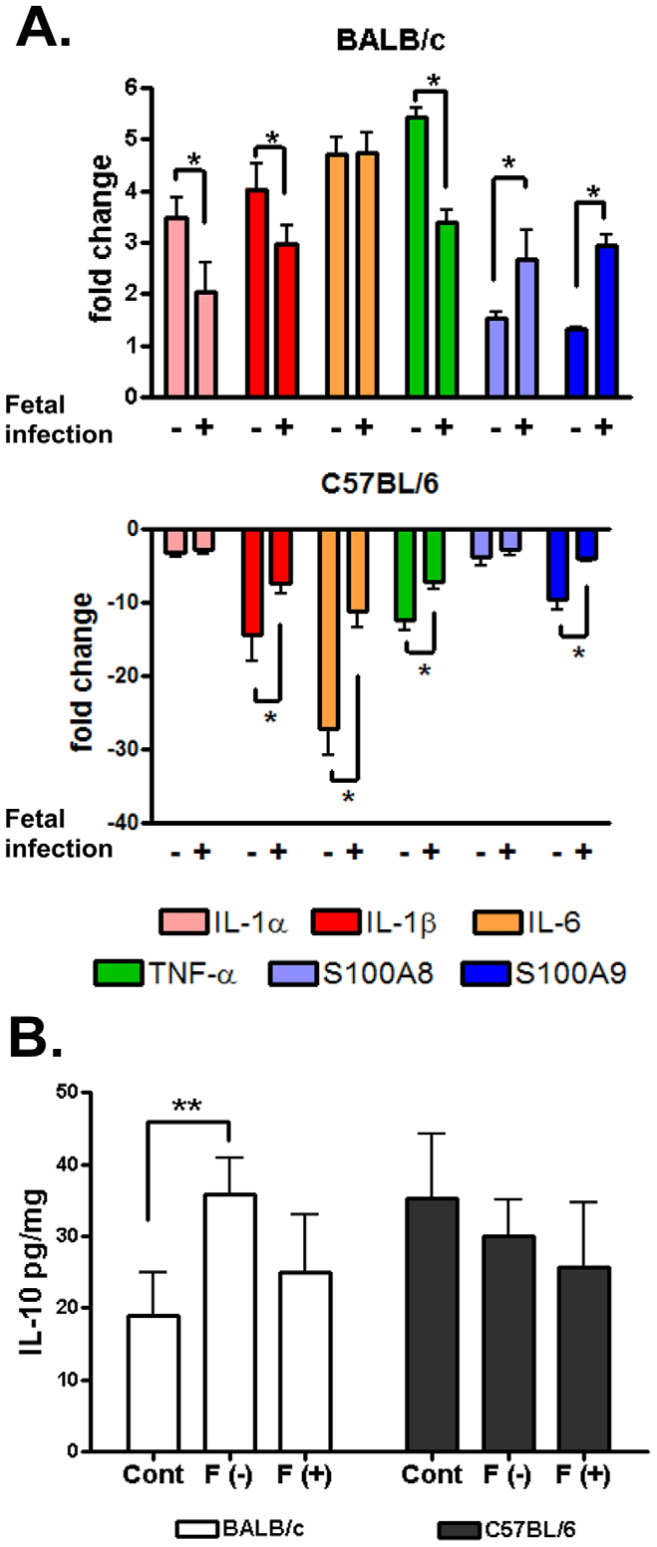
Placental inflammatory profiles in *U. parvum* infected BALB/c and C57BL/6 mice. ( A) Fold change (2*^−^* ^ΔΔ*Ct*^ ) is relative to normalized Ct values from sham inoculated controls, which was determined by the method of Schmittgen and Livak [72]. Values represent the mean 2*^−^* ^ΔΔ*Ct*^ ± SD of 5 biological replicates from 3 independent experiments.* Indicates a significant difference (P<0.01), which was determined by Student’s t test using 2*^−^* ^Δ*Ct*^ data. (B) Mean IL-10± SD expressed as pg/gm weight of tissue of 5 biological replicates from 3 independent experiments. ** Indicates a significant difference (P<0.03).

In order to determine if fetal infection impacted placental inflammation, we subdivided *U. parvum* infected placentas into fetus culture positive and fetus culture negative groups. *U. parvum* induced divergent placental gene expression profiles in BALB/c and C57BL/6 mice as shown in Figure 6A. Overall, *U. parvum* infection produced a pro-inflammatory profile in BALB/c mice. For example, in the BALB/c fetus culture negative group, *U. parvum* placental infection increased the expression of all pro-inflammatory mediators except for S100A9 (P<0.02). The production of IL-10 in *U. parvum* infected BALB/c placentas from fetus culture negative animals was also increased (Figure 6B, P<0.05). Although the BALB/c fetus culture positive group also displayed a significant increase in all pro-inflammatory mediators (P<0.01), the pattern of the change was different when compared to the fetus culture negative group. Specifically, fetal infection resulted in attenuated placental expression of IL-1α, IL-1β, and TNF-α, but the expression of S100A8 and S100A9 was augmented (P<0.005). Fetal infection also attenuated the production of placental IL-10 (Figure 6B). The degree of placental IL-6 gene expression was not affected by fetal infection in BALB/c mice. *U. parvum* infection induced an opposite effect in C57BL/6 mice. In this strain, placental expression of IL-1α, IL-1β, IL-6, TNF-α, S100A8, and S100A9 were all significantly decreased in *U. parvum* infected placentas obtained from uteroplacental units that had uninfected fetuses (P<0.001). Placental tissues from infected fetuses displayed a similar effect, but the degree of IL-1β, IL-6, TNF-α, and S100A9 suppression was significantly reduced when compared to the fetus culture negative group (P<0.05). In C57BL/6 mice, fetal infection did not affect the expression of IL-1α or S100A8 in *U. parvum* infected placentas. *U. parvum* infection did not have any impact on the production of placental IL-10 in C57BL/6 mice.

## Discussion

Ureaplasmas are one of the most common microbes isolated from the amniotic fluid of women during mid-trimester, but only a subset of women with positive cultures develop adverse pregnancy outcomes such as PTL, PROM, fetal infection and fetal inflammatory response syndrome [49], [56], [57]. The pathogenesis of ureaplasmal associated adverse pregnancy outcomes is further complicated by the fact that most placental infections involving ureaplasmas are polymicrobial [55], [58]. However, the impact of mixed bacterial infection on ureaplasmal pathogenesis is not clear since the inflammatory phenotype associated with pure ureaplasmal infections is indistinguishable from mixed bacterial infections that contain *Ureaplasma* [55]. Since an integral part of ureaplasmal associated disease involves a robust host inflammatory response, it has been suggested that the host’s reaction to the presence of the microbe is a major determinant in the development of adverse pregnancy outcome [31]. By using genetically distinct mouse strains with divergent innate immune responses, we have provided evidence that supports this theory (summarized in Table 3). Despite the fact that both C57BL/6 and BALB/c mice had equivalent placental microbial load, only the BALB/c mouse exhibited a greater proportion of fetal infection. Further, a striking feature in the BALB/c mouse was the presence of extensive neutrophilic infiltrates that were not present in the C57BL/6 mouse. This prevalence of neutrophils within the tissues of BALB/c mice may account for the increased rate of fetal infection along with the greater range of fetal pathology that was observed in this strain.

**Table 3:** Predominant BALB/c and C57BL/6 response profile to *U. parvum* intrauterine infection.

Phenotype	BALB/c	C57BL/6
Placental infection	Susceptible	Susceptible
Fetal infection	Susceptible	Resistant
Maternal inflammatory response	Moderate to severe	Minimal to moderate
Fetal pathology	Multi-organ	Intestine and liver

Our experimental approach was designed to elicit both maternal and fetal inflammatory responses and to ensure a high rate of placental and fetal infection. Therefore, a high dose of *U. parvum* was deposited into the uterine lumen instead of the amniotic cavity, which allowed for microbial dissemination that resembled ascending infection. As supported by our data, both mouse strains developed high placental infection rates (greater than 90%) that contained microbial loads (CFU of 10^3^ or greater) reported to correlate with intrauterine inflammatory responses such as chorioamnionitis, PROM, fetal morbidity and mortality in humans [12]. Immunodetection of *U. parvum* demonstrated that the *in situ* distribution of the microbe within the placenta, amniotic cavity, and fetal intestine was also the same in both mouse strains. Therefore, the different rates of fetal infection between C57BL/6 and BALB/c mice cannot be attributed to differences in microbial load.

Since our experimental approach was designed to mimic ascending infection, we cannot exclude the possibility that there may have been some variation in the timing of intra-amniotic invasion among the fetal units within each uterine horn. However, our experimental design adjusted for this potential variation in several ways. First, inherent variations were blocked in our analysis by using all fetal-placental tissues within each uterine horn, including those farthest from the inoculation site. Second, studies in our laboratory have demonstrated that the rate of *U. parvum* spread to all fetal units within the uterus is rapid. For example, a pilot study that included 7 C57BL/6 dams showed that 91% of placentas and 33% of fetuses were colonized with *U. parvum* by 24 hours post-inoculation (unpublished data). Therefore, analysis of tissues at 72 hours post-inoculation provided sufficient time for adequate microbial dissemination into each feto-placental unit, which was confirmed by a placental colonization rate of 100% in C57BL/6 mice. Interestingly, we observed a higher rate of fetal infection in our pilot study (45% at 24 hours vs. 18% at 72 hours), which implies that some microbial clearance may be occurring in C57BL/6 mice as the duration of infection increases.

A striking feature in BALB/c dams was the extensive neutrophilic infiltration that was present in both uterine and placental tissues. Most neutrophils in BALB/c tissues appeared to be undergoing necrotic death instead of apoptosis. Necrotic death prolongs neutrophil activation and inflammation, delaying neutrophil clearance and the reparative response [59]. Other features that support ongoing inflammation in BALB/c tissues included increased expression of pro-inflammatory mediators, thickening of the amniotic basement membrane, and extensive desquamation of epithelial cells. Taken together, these features are consistent with prolonged inflammation of at least 36 to 48 hours and with breach of the maternal-fetal barrier [32], which may have contributed to the increased rate of fetal infection observed in this mouse strain.

The divergent placental inflammatory responses that were observed in C57BL/6 and BALB/c mice may be due to inherent differences in their neutrophil function, toll-like receptor (TLR) responsiveness [45], [50], and/or macrophage dominant profiles [46], [50]. These host specific factors can impact disease outcome independently of *U. parvum* mediated effects on the host. For example, Allenbach et al [42] demonstrated that C57BL/6 neutrophils undergo extrinsic apoptosis during the early phase of *L. major* infection, which is important for reducing the damaging effects of inflammation during infection with this pathogen. In our study we observed scant influxes of neutrophils in *U. parvum* infected uterine and placental tissues of C57BL/6 mice without evidence of extensive tissue necrosis, which may be due to non-reactive neutrophil clearance [59] in this mouse strain. Further, C57BL/6 mice exhibited higher levels of placental IL-10 production that did not significantly dissipate in the presence of infection. This would also support resolution of inflammation and a reparative response [59]. Conversely, BALB/c mice infected with *L. major* have defective extrinsic neutrophil apoptosis coupled with severe protracted neutrophilic inflammation [42], [43], which is similar to what we observed in *U. parvum* infected tissues of this mouse strain. BALB/c mice also have decreased responsiveness to TLR agonists or cytokines [45], [50], and typically produce a suppressive or macrophage M2 dominant immune response [44], [46], [50]. Specifically, macrophages from BALB/c mice typically produce prostaglandin E_2_ (PGE_2_) when exposed to lipopolysaccaride, which inhibits a TH1 cytokine response in these animals [44]. BALB/c mice also produce less TNF-α and IL-12 in response to MALP-2, which is a mycoplasma protein that is a TLR2/6 specific agonist [45]. Compared to C57BL/6 mice, placental tissues of sham inoculated BALB/c mice exhibited decreased expression of IL-1β, IL-6, and TNF-α, which is more in tune with M2 macrophage polarization [60]. This feature may be particularly critical in adverse pregnancy outcomes involving ureaplasmas since ureaplasmas elicit PGE_2,_ IL-1β, TNF-α, and IL-10 from human amniochorion and choriodecidual explants [61], [62], which is neither characteristic of a TH1 or TH2 cytokine response. Thus, *U. parvum* infection in the M2 dominant BALB/c mouse may trigger an attenuated innate response that is not microbicidal [45], but instead results in a detrimental inflammatory response.

The fetal inflammatory response profiles observed in C57BL/6 and BALB/c mice also implied that the extent of fetal infection was different in these strains. For example, pathology was only detected in the liver and intestine of C57BL/6 fetuses, and *U. parvum* was only identified in the intestinal lumen of these animals by IFA. This would suggest that infection was localized and most likely occurred by fetal ingestion of contaminated amniotic fluid. On the other hand, the presence of varying degrees of chorionic vasculitis and/or umbilical vasculitis [3], [32], extensive influx of neutrophils into the amniotic cavity, coupled with multiple fetal organ pathology (brain, lung, heart, liver and intestine) in BALB/c fetuses was more consistent with fetal sepsis and fetal inflammatory response syndrome [2], [63], [64]. Other indicators of fetal sepsis and/or fetal inflammatory response syndrome were elevated IL-6 and S100A8 in placental tissues [63], [64]. Although hepatic lesions observed in *U. parvum* infected fetuses is not a feature of fetal inflammatory response syndrome, hepatic lesions have been observed during human fetal infection with *Ureaplasma* species [65], [66] as well as in the sheep model of *U. parvum* intra-amniotic infection [67].

Limitations with this study include 1) lack of earlier time points that would elucidate the temporal progression of maternal and fetal inflammatory responses during microbial spread into the amniotic cavity, 2) clear distinction of fetal leukocytes from maternal leukocytes within the amniotic cavity, and 3) the lack of fetal organ specific cultures that would have clarified if BALB/c fetuses indeed had multiple organ infection and sepsis. Despite these limitations, this study demonstrated that the host genetic background significantly impacts disease outcome during intrauterine infection with *U. parvum.* Although our study cannot conclude that similar effects may be observed with intrauterine infections caused by other microbial species, it does underscore the need to consider the host genetic background as a factor in disease pathogenesis, especially studies that use the mouse as an infection model.

## Materials and Methods

### Animals

Breeding colonies of C57BL/6 and BALB/c strains were established with specific-pathogen-free animals purchased from the Jackson Laboratory (Bar Harbor, Maine). Heath reports can be accessed at http://jaxmice.jax.org/list/ahname.html. All animals were handled in accordance with procedures approved by the University of Florida Institutional Animal Care and Use Committee. Animals were handled and maintained under barrier conditions at all times. Syngeneic breedings were performed with 10–16 week old primiparous females using a harem breeding system. Successful breeding was determined by the presence of a copulatory plug, which was considered gestation day of 0. Sham inoculated control animals and animals inoculated with *U. parvum* were housed singly in static caging under ABSL-2 conditions.

### *In vivo* passaging of *U. parvum*

A strain of *U. parvum* that was previously adapted in Sprague Dawley rats [68] was *in vivo* passaged 5 consecutive times in both BALB/c and C57BL/6 mice in order to adapt the strain to this species. Briefly, at each passage, one to two pregnant mice were intravenously inoculated with 10^5^-10^9^ CFU of *U. parvum* into the lateral tail vein at gestation day 14. An additional inoculum was also instilled into the vaginal cavity of each animal. Vaginal cultures were collected at 24 h post-inoculation (PI). At 48 h PI, animals were euthanized, and vagina, uterus, and fetal-placental units were cultured for *U. parvum*. A *U. parvum* isolate that was isolated from a fetus and also grew well in cell free media was selected for future experiments. This isolate was expanded in a second in vitro passage, which became the working stock for subsequent experiments.

### *U. parvum* Preparation and Culture

For all experiments, *U. parvum* was cultured and processed as previously described [68], [69]. For infection studies, 1 ml of the working stock was grown in 45 ml of 10B broth to early stationary phase at 37°C. The CFU of each inoculum was determined by optical density measurement and confirmed by culture. Cultivation of *U. parvum* from inoculates and mouse tissues were performed as previously described [68]. Briefly, both color changing units and colony forming units (CFU) were determined for each sample. However, a sample was only considered positive if *U. parvum* colonies were identified on A8 agar.

### Experimental Intrauterine Inoculations

In order to avoid cross contamination sham controls were inoculated before infected animals and always handled before infected animals for the duration of the study. Animals received intrauterine inoculations of sterile vehicle (control) or 10^7^ CFU of *U. parvum* at gestation day (GD) 14 in order to mimic ascending infection. Surgical procedures were performed under a class 2 laminar flow hood using aseptic technique. Briefly, the ventral abdomen was shaved, and the incision site was prepared aseptically with alternating chlorhexidine acetate 2% (w/v) scrub (Nolvasan®, Fort Dodge) and warm sterile saline. Anesthesia was induced and maintained with Isothesia (Butler Shein Animal Health, Dublin, OH). A 1–2 cm ventral midline incision was made, the proximal portions of the horns exteriorized, and 100 µls of inoculum was injected into the uterine lumen of each horn between the cervix and the first fetus of each horn. Non-pregnant horns were not inoculated. Muscle and subcutaneous layers were closed with absorbable suture and skin was closed with stainless steel wound clips (Autoclips®, Harvard Apparatus). For perioperative support, animals received 1 ml warm normal saline subcutaneously (SC) for fluid replacement and buprenorphine hydrochloride for analgesia (Carpuject, Hospira, Inc., Lake Forest, IL) administered subcutaneously 0.05–0.1 mg/kg every 12 hours for 48 hours starting preoperatively. For each experiment, a minimum of one mouse from each strain was inoculated.

### Necropsy

After euthanasia the abdomen was sprayed with 95% ethanol and opened. Abdominal organs were inspected for gross lesions and the uterus was aseptically removed for processing. Six fetal units from each dam were processed for gene expression analysis, microbial culture, and histopathology. Each fetal unit was assigned an accession that corresponded to its placenta so that placental pathology, *U. parvum* culture status, and placental gene expression could be correlated to fetal infection status. Briefly, the fetus was aseptically removed into a separate culture dish; the skin was disinfected with 70% ethanol before being cultured for *U. parvum* as already described [70]. Each placenta was then transected in half so that one half was used for histopathology and the other half was processed for gene expression analysis or *U. parvum* culture. Tissues saved for gene expression analysis were immediately frozen in liquid nitrogen and stored at -80°C until further processing. Remaining fetal/placental units were processed *in toto* for histopathology and *in situ* detection of *U. parvum* by IFA.

### *In situ* Detection of *U. parvum*

Paraffin embedded formalin fixed tissues were processed as previously described [69], [71] with the one exception that antigen retrieval was performed with heat using sodium citrate buffer [10 mM Sodium citrate, 0.05% Tween 20, pH 6.0].

Images were captured with an Olympus IX81-DSU Spinning Disk confocal microscope using Slidebook software (Olympus, Center Valley, PA). During image capture, camera settings were optimized using the tissue section with the highest degree of fluorescent signaling. Once the image capture settings were established, the same settings were used for all the tissues within the specific labeling experiment**.** Isotype control images are provided in supporting file S1.

### Histopathology

Tissues were fixed in 10% buffered formalin for 24 hours then transferred to 95% ethanol until processing. Tissues were trimmed and transected so that a cross-sectional view of the placenta and associated endometrium and chorioamnion would be present. Tissues were processed routinely and stained with hematoxylin and eosin.

In order to develop a lesion scoring system, the pathologist was aware of which samples came from control animals but was blinded as to identity of mouse strain. After development of the lesion scoring system, all slides were recoded so that the pathologist was blinded to mouse strain and experimental treatment (infection status). The scoring system for chorioamnionitis was based on criteria previously established and validated for human placental tissues [3], [32]. Our modified scoring system for evaluating maternal and fetal inflammatory responses is summarized in Tables 1 and 2.

### Placental Inflammation Profiling by Quantitative RT-PCR or ELISA

Mouse placental tissues were selected for analysis on the basis of treatment (control vs. infected) and fetal culture status (positive or negative for *U. parvum*). At least 5 biological replicates from each group were analyzed. Total RNA from mouse placental tissues was extracted with TRIzol (Life Technologies™) according to the manufacturer’s instructions. Quantification of TNF-α, IL-1α, IL-1β, IL-6, S100A8, S100A9 and GAPDH mRNAs were determined by SABiosciences QPCR primer assays with SYBR green detection (Qiagen, Valecia, CA). All PCR reactions were performed according to the manufacturer’s instructions on an iCycler-IQ, version 3.1 using Optical System Software 3.1 (BioRad Laboratories, Hercules, CA). Cycle threshold (Ct) values from each sample were normalized with its corresponding GAPDH Ct. Normalized values were converted to log base 2 before data was evaluated by statistical analysis [72].

Placental tissues were homogenized in T-PER protein extraction reagent (Pierce Biotechnology, Inc) that was supplemented with HALT protease inhibitor cocktail according to manufacturer’s instructions. Placental protein extracts were analyzed for the presence of mouse IL-10 by ELISA (Pierce Biotechnology, Inc.) according to manufacturer’s instructions. IL-10 concentrations were normalized by mg placental tissue weight.

### Statistical Data Analysis

Data from multiple experiments were grouped together in order to make statistical analysis possible. When analysis involved more than 2 comparisons, data were analyzed by one-way analysis of variance (ANOVA) followed by Fisher’s multiple comparison test if ANOVA indicated a significant difference among group means. Unpaired Student’s T test was used for comparisons between 2 groups. Nonparametric data was analyzed by Kruskall-Wallis or Chi square analysis. For all analyses a probability of P≤0.05 was considered significant.

## Supporting Information

File S1 **Isotype control images are provided.**
(TIF)
