# Supplementary figures and images for: Host Genetic Background Impacts Disease Outcome During Intrauterine Infection with Ureaplasma parvum
Source: PLoS One. 2012 Aug 29;7(8):e44047. doi: 10.1371/journal.pone.0044047 (PMC3430619; doi:10.1371/journal.pone.0044047)

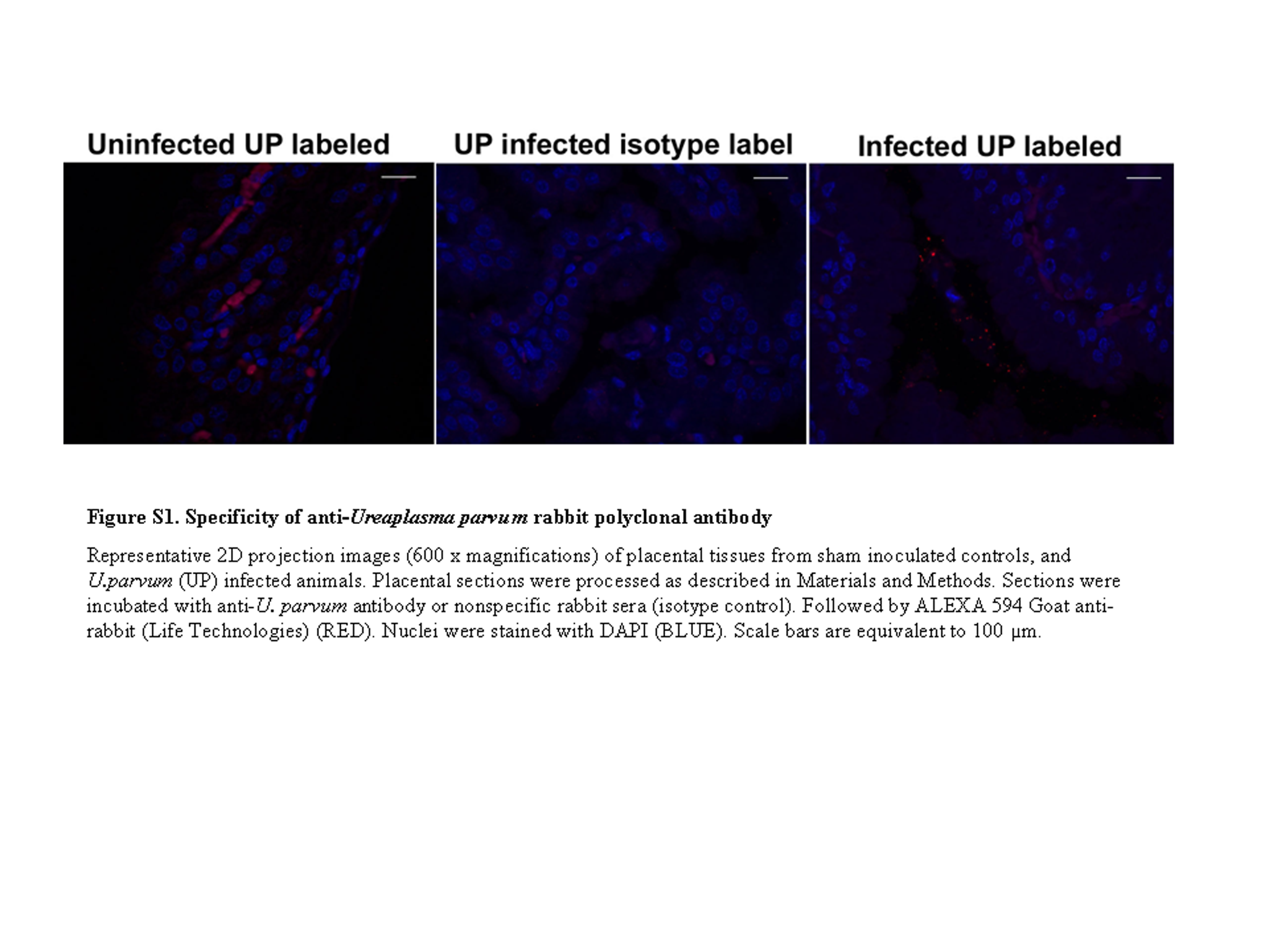

Supplement: File S1 — Isotype control images are provided. (TIF) [file pone.0044047.s001.tif]
